# Implementation of QbD Approach to the Development of Chromatographic Methods for the Determination of Complete Impurity Profile of Substance on the Preclinical and Clinical Step of Drug Discovery Studies

**DOI:** 10.3390/ijms231810720

**Published:** 2022-09-14

**Authors:** Lidia Gurba-Bryśkiewicz, Urszula Dawid, Damian A. Smuga, Wioleta Maruszak, Monika Delis, Krzysztof Szymczak, Bartosz Stypik, Aleksandra Moroz, Aleksandra Błocka, Michał Mroczkiewicz, Krzysztof Dubiel, Maciej Wieczorek

**Affiliations:** Celon Pharma S.A., ul. Marymoncka 15, 05-152 Kazuń Nowy, Poland

**Keywords:** Analytical Quality by Design (AQbD), design of experiment (DOE), method operable design region (MODR), pharmaceutical impurity profiling, analytical method development, CHI logD, CPL409116, JAK/ROCK inhibitor

## Abstract

The purpose of this work was to demonstrate the use of the AQbD with the DOE approach to the methodical step-by-step development of a UHPLC method for the quantitative determination of the impurity profile of new CPL409116 substance (JAK/ROCK inhibitor) on the preclinical and clinical step of drug discovery studies. The critical method parameters (CMPs) have been tested extensively: the kind of stationary phase (8 different columns), pH of the aqueous mobile phase (2.6, 3.2, 4.0, 6.8), and start (20–25%) and stop (85–90%) percentage of organic mobile phase (ACN). The critical method attributes (CMAs) are the resolution between the peaks (≥2.0) and peak symmetry of analytes (≥0.8 and ≤1.8). In the screening step, the effects of different levels of CMPs on the CMAs were evaluated based on a full fractional design 2^2^. The robustness tests were established from the knowledge space of the screening step and performed by application fractional factorial design 2^(4−1)^. Method operable design region (MODR) was generated. The probability of meeting the specifications for the CMAs was calculated by Monte-Carlo simulations. In relation to literature such a complete AQbD approach including screening, optimization, and validation steps for the development of a new method for the quantitative determination of the full profile of nine impurities of an innovative pharmaceutical substance with the structure-based pre-development pointed out the novelty of our work. The final working conditions were as follows: column Zorbax Eclipse Plus C18, aqueous mobile phase 10 mM ± 1 mM aqueous solution of HCOOH, pH 2.6, 20% ± 1% of ACN at the start and 85% ± 1% of ACN at the end of the gradient, and column temperature 30 °C ± 2 °C. The method was validated in compliance with ICH guideline Q2(R1). The optimized method is specified, linear, precise, and robust. LOQ is on the reporting threshold level of 0.05% and LOD at 0.02% for all impurities.

## 1. Introduction

The pharmaceutical industry is closely regulated by the quality control system. The quality of pharmaceutical products and substances (API) should be assured and documented at every stage of the manufacturing and product lifecycle. According to the International Council for Harmonization ICH Q9 guideline [1], it is not enough to ensure quality, it is also necessary to identify and control the potential quality problems arising during the process of development and manufacture. In order to fulfil this requirement, a systematic approach to the pharmaceutical process and product development is necessary. This approach is known as quality by design (QbD) and is outlined in the ICH Q8 guideline [2]. Originally, the QbD methodology focused on the development of pharmaceutical manufacturing but recently it has been implemented into the development and optimization of analytical methods and is called Analytical Quality by Design (AQbD) [3,4,5,6,7,8].

Appropriate analytical methods are necessary to ensure the effectiveness and safety of a pharmaceutical product and to meet regulatory requirements, as well as QC needs. The inefficient analytical methods can lead to inaccurate results and misleading information, significantly affecting the drug development process. The application of the AQbD approach ensures that analytical procedures are well understood, robust, and consistently deliver the intended performances throughout their lifecycle.

The application of QbD into analytical methods development has been well adopted by the pharmaceutical and biopharmaceutical analysts [3,4,5,6,7,8,9,10] and has progressively increased over the last years to achieve a higher quality of analytical methods and thus a higher quality of the pharmaceutical product. Most of the applications are focused on the use of experimental design (DOE) and statistical screening of spaces of the method’s operating parameters to account for method robustness, especially for separation techniques [11,12,13,14,15,16,17,18,19,20]. Also, there have been published papers describing a general, systematic life-cycle approach to the development of analytical procedures. [6,8,21,22,23,24,25]. However, much more effort is still required to improve the AQbD procedures and target the concept towards all kinds of methods from clinical to commercial and manage their lifecycle management as well as updated regulations. Therefore, it is expected that the update of ICHQ2(R1) [25] and the development of a new ICHQ14 [26], guidelines for the development of current analytical procedures will be released soon and is an excellent opportunity to define how analytical methods should be developed, described, and validated in regulatory submissions.

**Figure 1:**
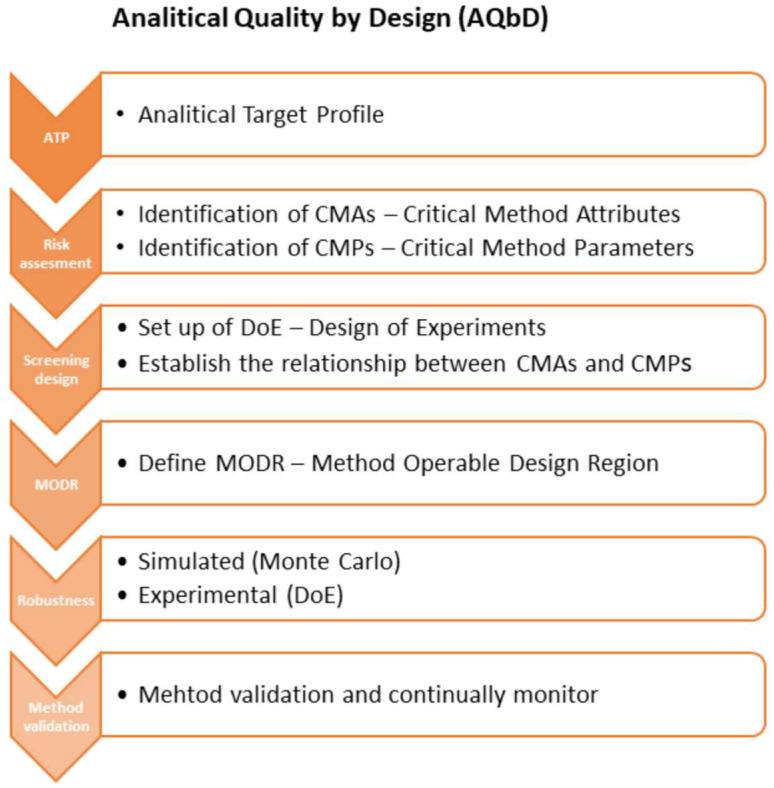
AQbD methodology workflow on the base of Tome at al. [27].

The AQbD methodology is quite similar to the QbD process as described in ICH Q8 [2]. The starting point is to define the intended purpose of the method and the analytical target profile (ATP), while in QbD it is the step to define the quality target product profile (QTPP). Each analytical method should have its intended purpose, taking into account the area of its application, for example, to support research, manufacturing process, formulation development, release, and stability testing for clinical or commercial drugs, quantitative, qualitative, or limit tests. The method ATP is determined by combining the intended purpose and the ICH Q2(R1) quality requirements [25], such as specificity, precision, accuracy, linearity, range, quantitation limit, and detection limit. Furthermore, the potential critical method attributes (CMAs) could be determined as an analytical equivalent of the critical quality attributes (CQAs) in the QbD approach. CMAs should fulfil the specification requirements as well as the quality limits of the measured parameters. The next step of AQbD workflow is risk-assessed to identify and prioritize factors (critical method parameters, CMP), that can affect the method attributes [11,19,24]. The Ishikawa diagram is a useful tool to identify critical method parameters. CMPs are further investigated for robustness using the statistical DOE and multivariate analysis. DOE is a structured, cost-effective, and cost-efficient method to organize, limit the number of experiments, and determine the simultaneous effects and interactions of multiple CMPs on the CMAs. The objective of DOE is the definition of the analytical DS—MODR (method operable design region), which is the operating range of CMPs that guarantee quality results. [9,20,21]. Figure 1 shows the scheme of the AQbD methodology.

**Figure 2:**
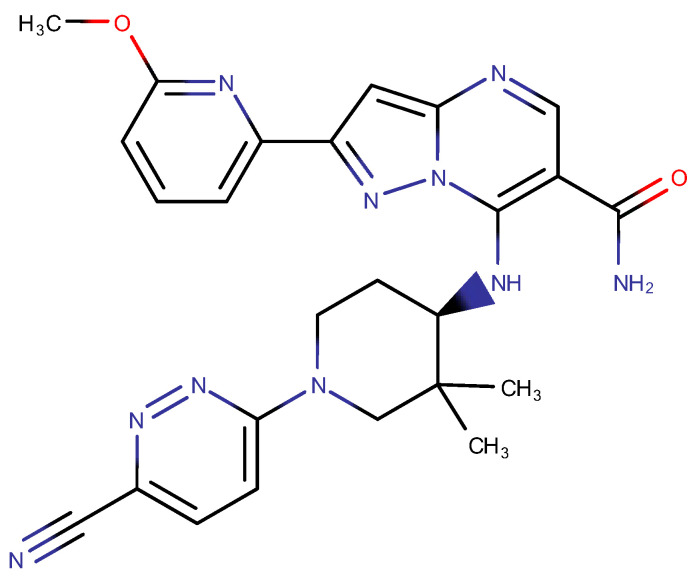
Chemical structure of CPL409116.

The aim of the study was to apply the AQbD approach to the methodical step-by-step development of a chromatographic method for determining the full profile of impurities, process, and degradations, an innovative pharmaceutical substance of the CPL409116 (Figure 2), dual JAK (Janus kinase) and ROCK (Rho-associated kinase) inhibitor [28,29,30].

Control of the purity of the CPL409116 substance was crucial at the stage of development of the process of large-scale synthesis and the production of the substance for preclinical and clinical trials.

To meet the quality requirements of the formal ICH regulatory guidelines [2,25], but bearing in mind the cost and time effectiveness of the preclinical stage of drug development main goal was to design a quick, simple, and robust analytical method that allows easy application for control of manufacturing of finished dosage form of a pharmaceutical product.

Based on our best knowledge, the literature presented works related to the partial introduction of QbD to the correction, verification, and improvement of pharmacopeial methods or methodologies of known pharmaceutical substances [11,12,19,31,32]. Our studies were focused on the implementation of the complete AQbD approach including screening, optimization, and validation steps, for the development of a new method for the quantitative determination of the full profile of nine impurities from the process and degradation of an innovative pharmaceutical substance. In our case, structure-based analysis was a very important step due to the greater differences in the structures of the tested compounds than in the case of the presented optimizations of the methods for the separation of related compounds. For these reasons, the structure-based pre-development studies based on the calculated physicochemical properties such as logP, logD, pKa including CHI logD—experimentally determined chromatographic hydrophobicity index were also performed.

Our work concerns a wider range of simultaneously optimized parameters of the chromatographic method compared to those described in the literature. The development presented in [31,32,33] was carried out on one column, while our study tested eight different stationary phases. We also used a much wider screening of pH values of the aqueous mobile phase (from 2.6 to 6.8).

Moreover, apart from the commonly considered resolution between neighbors peaks as a critical method attribute (CMA), we also took into account the peaks symmetry factor, which is important in terms of quality criteria.

## 2. Results and Discussion

### 2.1. Analytical Target Profile (ATP)

Controlling the impurity profile of a pharmaceutical active substance and pharmaceutical formulation is a key element for drug development and quality assurance since the provided data can directly influence the safety of drug therapy, reducing the impurity-related adverse effects of drug products.

There are two main steps of the control of impurities during the manufacture of drugs, the control of raw materials before drug manufacturing and the control of finished products before batch release. This two-stage control process requires selective, specific, and sensitive analytical techniques. In this context, the most current techniques coupled to AQbD for the quantitation of impurities in active pharmaceuticals ingredients (APIs) are high-performance liquid chromatography (HPLC) and ultrahigh-pressure liquid chromatography (UHPLC) in reversed-phase mode with UV or mass spectrometer detectors [9,11,12,14,15]. In this work, the UHPLC method with UV detection was optimized.

The first step in the AQbD approach is to define ATP [11,12,13]. The ATP conceptualizes the objective of the analytical method, but also the quality requirements for the reportable result, which include both performance characteristics associated with one or more CMAs and criteria for validation parameters for demonstrating that the method is fit for purpose [11,20]. When dealing with quantitative determination of impurities, the ATP is mainly focused on method selectivity to ensure a complete separation between API, related and unknown impurities, and at a pinch also—excipients. The second objective is also to achieve the required sensitivity of the method (limit of quantitation (LOD) equal or lower than the 0.05%) [11,13]. The method under development was intended to be used in routine quality control, hence the criteria for ATP related to the validation parameters were generally taken from the ICH Q2 (R1) guidelines [9,25].

Therefore, in the present study, ATP is set as: (a) a robust, selective, and specific method, (b) good linearity, precision, and accuracy of impurities determination, and (c) statistically challenged validation [11,12].

### 2.2. Risk Assessment, Critical Method Attributes CMAs, Critical Method Parameters CMPs

**Figure 3:**
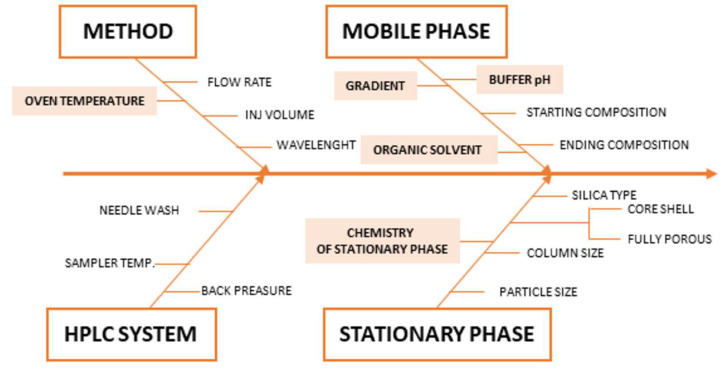
Ishikawa diagram for critical method parameters (CMP).

CMAs are an element of method performance that must be measured to access whether a method can produce fit for purpose data. The CMAs are directly connected with the ATP and are response variables that can give information on the quality of the chromatogram. In the present study, the CMAs are: (a) resolution between the peaks should be ≥2.0, and (b) peak symmetry of analytes should be ≥0.8 and ≤1.8. Many parameters could influence the chromatographic performances (of CMAs), which involve aspects related to the chromatographic system, the column, and the mobile phase. In order to indicate the risk factors of the chromatographic method, a fishbone diagram was performed as shown in Figure 3 [11,14,34].

From this diagram, it was possible to identify the CMPs that could potentially affect the selected CMAs and, therefore, required an in-depth investigation using DOE methodologies. The following parameters were considered for the multivariate optimization: stationary phase chemistry, pH of aqueous eluent of the mobile phase, start and stop percentage of organic eluent of the mobile phase, and oven temperature.

The settings of the other factors were selected based on preliminary experiments. In particular, following common practice, the injection volume was set to a low value (1 μL), gradient time was set to 7 min (to obtain optimum analysis time, elution time for all of the compounds is lower than 4 min), and the flow rate of mobile phase was set to 0.5 mL min^−1^.

In this study linear gradient elution has been chosen. This is simple and typically the default elution method (“first choice” method) during chromatography methods development. The linear gradient minimizes peak broadening against isocratic elution and produces more accurate and repeatable results. This second factor is particularly relevant when a transfer method to another instrument is an option, thus linear elution allows for the development of a more universal methodology. Different gradient speeds have occurred by changing the initial and final amount of organic phase, at a range from 20% to 25% and from 85% to 90%, respectively.

ACN was chosen as a type of organic solvent in the mobile phase. MeOH as an organic solvent has been disregarded on a base of preliminary studies, due to high retention and no elution of some of the tested compounds.

Moreover, the wavelength of UV detection was determined and set to 297 nm as a maximum of absorbance JAK01 (CPL409116) and eight of its impurities and 230 nm to control the JAK SM-05 impurity.

### 2.3. Structure-Based Pre-Development Study

One of the first steps during the development of a chromatographic method is to a knowledge of the chemical structure of analyzed compounds to assess opportunities and identify initial risks during optimization of the analytical method, and also to determine correctly CMPs, like pH of the mobile phase. The basic molecular descriptors, which strongly affect the retention factors, are hydrophobicity (lipophilicity) of molecules or ions, expressed by the partition coefficient logP, and distribution coefficient (logD) for ionizable compounds [35,36,37]. In addition, most of the new drug molecules that are currently undergoing preclinical research or clinical trials contain ionized groups. Acid-base chemistry strongly influences not only retention and chromatography resolution but also affects band broadening [38,39], which further complicates the development of an effective separation method.

For the initial characterization and determination of the differences between the CPL409116 (JAK01) compound and its impurities, calculations of the physicochemical properties (logP, logD, and pKa) have been performed using the ACD Labs Percepta program (Table 1) [40]. On this basis, it was found that most of the molecules have basic dissociation constants pKa in the range 1.98–2.70 except for JAK-07 (0.90 and 3.98) and JAK-08 (0.79 and 2.70), and JAK ImpB characterized by basic pKa 2.70 and 3.56, but in particular, JAK-09, having a different basic nature (pKa = 2.68 and 9.92). In the pH range of 5.5–8.0, all compounds exist in a single, unprotonated form, which definitely facilitates the development of the chromatographic method.

A much more complicated situation was concerned with the descriptors determining the lipophilicity of compounds (logP and clogD). Generally, logP factors show a promising distinction of retentions in chromatographic conditions. But the data obtained for the compound JAK ImpC (logP/logD = −4.22) suggested very poor retention that was inconsistent with the observed retention times of the compound in chromatographic conditions.

Due to this observation and to better characterise elution conditions for method development, it was decided to determine the chromatographic hydrophobicity index CHI logD of CPL409116 and its impurities by the methodology described by Valko et al. [41]. Chromatographic hydrophobicity index CHI logD has been originally used for high-throughput physicochemical property profiling for rational drug design [42], where determined values strongly correlate to lipophilicity (logP and/or logD) of molecules [37,41].

On a basis of determining lipophilicity described by CHI logD at pH 2.6, 7.4 and 10.5 on ACE Ultracore SuperC18 column more valuable data were obtained in a contest of chromatography interactions molecules with stationary phase extended with their acid-base properties (Table 1). Particularly, strong pH dependence on retention factors has been observed for JAK ImpC and also for JAK07, JAK08, and JAK09. Greater differences and a wider range of CHI logD values, calculated on a base of peaks retention times of compounds, in the lower pH range than in the pH 7.4–10.5 range have been observed. The use of mobile phases with a lower pH would allow obtaining higher chromatographic resolutions and higher selectivity of the analytical method.

Moreover, CPL409116 is practically insoluble in aqueous solutions (e.g., in phosphate buffer with a pH of 7.4 below 0.1 µg ml^−1^), hence the choice of more acidic separation conditions where the compound may protonate is expected to reduce carryover, problems with clogging of the column and its longer lifetime.

In summary, various dissociation constants of the tested compounds made it necessary to check the elution conditions in more detail in the pH range 2.6–6.8, taking into account peak shape. Using more acidic conditions in the mobile phase should lead to the preparation of more robust and effective analytical methods. As a final CMP, six different buffers at pH 2.6, 3.2, 3.8, 4.0, 4.2, and 6.8 as an aqueous mobile phase has been chosen.

Impurities generally have molecular structures that are close to or related to the API, they also tend to show similar chromatographic behavior. In the case of impurities of CPL409116 JAK ImpC and JAK ImpA have very close hydrophobic index CHI logD at pH 2.6 that same like main compound have similar retention to JAK07 or JAK ImpD and JAK ImpE at pH 7.4 (Table 1).

It is possible to modulate the effectivity of separation and chromatographic resolution by changing the pH of the mobile phase or temperature. But a much more effective way is modulation of selectivity of stationary phase that is strongly important if analysed compounds have a very close molecular structure with comparable hydrophobicity. In this manner, the most effective idea is to explore the selectivity space and chemistry of stationary phases. Based on the work of Snyder and Dolan [43], [44] five terms describing selectivity are: (a) the hydrophobic interaction between the solute and the column; (b) the resistance of the bonded phase to penetration by bulky molecules; (c) hydrogen bond (H-B) interactions between basic solutes and acidic sites (silanols) in the column; (d) H-B interactions between acidic solutes and a vicinal-silanol pair on the column surface; and (e) cation exchange between ionized bases and ionized silanol groups in the column. On this base the Hydrophobic-Subtraction Model (HSM) has been prepared for quantitative comparison of differences in chromatographic columns and the resulting data are publicly available for free through different websites, i.e., www.hplconline.org [45] (accessed on 29 January 2021 or USP chromatographic column database [46].

For the present work, eight columns that differ in chemistry and parameters of stationary phase have been chosen (Table 2).

Columns have been chosen on a base similarity factor Fs and also taking into account differences in acid-base interaction of stationary phase (C(pH 2.8) and C(pH 7.0). CPL409116 contains a few different heterocyclic rings, aliphatic amine functionality, amide, ether and nitrile substituents chemistry of stationary phases has been diversified between different chemical modifications, octadecyl, phenyl-hexyl, biphenyl, and pentafluorophenyl.

Therefore, during screening, the authors advise the fixing of qualitative factors such as the type of stationary and mobile phases based on scientific knowledge of the molecules and any relevant impurities. These parameters are very unlikely to change during routine use. Subsequently, quantitative factors such as pH, and the composition of the mobile phase or temperature, can be examined when optimizing a method for robustness. If necessary, pre-tests on qualitative factors can be conducted. Usually, the chosen columns will be those optimizing peak shapes, time of analysis, and selectivity. Knowledge of the interactions between the stationary phase, the mobile phase, and the molecule of interest should of course be included in such studies.

At the stage of preclinical and clinical development, a very important factor is the assessment of the potential genotoxicity and mutagenicity of new compounds in accordance with the requirements of the ICH M7 (R1) guidelines [47]. In the case of risk or analysis of mutagenic compounds, it is necessary to use very sensitive analytical methods, which in most cases means the use more sophisticated methodology or mass spectrometry detection [48,49].

Compound CPL409116 and their related substances, processing by-products and impurities, have been evaluated by two complementary (Q)SAR prediction methodologies to assess the potential mutagenicity of impurities. One methodology should be expert rule-based (DEREK Nexus, module in Star Drop software) [50,51,52] and the second methodology should be statistically-based (Toxicity Estimation Software Tool (T.E.S.T)) [53,54].

No structural alters and no mutagenicity risks were found for the CPL409116 and its related substances (Table 3) and it did not require changes to the previous assumptions regarding the level of impurity quantification.

### 2.4. Screening Study

The Screening Phase Method development was initiated with the screening activity using an Agilent scouting system equipped with an 8-position column manager and solvent selection valve. For screening experiments, eight column chemistries with a wide range of selectivity difference, wide pH range, different bonding, and with different similarity factors (Fs) [12,43] were used. A large difference in Fs factor indicates that the two columns are very different. During this study, columns’ Fs values were compared with Agilent ZORBAX Eclipse Plus C18. Columns selected, operating pH range of the columns, bonding details, and Fs values were mentioned in Table 2.

The buffer pH selection was carried out in the range of pH 2.6–6.8. Formic acid solution (10 mM) was used to prepare a buffer solution of pH 2.6, and ammonium formate (10 mM) buffer with different concentrations of additives was used to prepare buffer solutions of pH 3.2, 4.0, and 6.8. The initial screening experiments were carried out using different columns, different pH buffer solutions, and different components of a start (from 20% to 25%) and end gradient (from 85% to 90%) organic modifier compositions as variables.

The statistical experimental design was performed based on full fractional design 2^2^ (two factors: start and end gradient composition, two-level) with full repetition for each tested column and difference pH of buffer solutions [13,14]. Flow rate, gradient time, and column temperature were kept constant. A total of 128 experiments were performed by statistical design at the screening step.

After running the screening experiments, processing was done to integrate all the peaks properly and then the generated chromatographic responses were transferred to the STATISTICA software [55,56,57] to generate knowledge space for linear additive effects, curvilinear effects, and complex effects of different variables.

Multiple linear regression was applied for the calculation of the coefficients of the nine models for resolution between all pairs of peaks (R_s_) and 10 models of the symmetry factors for all peaks (A_s_), then the models were refined to improve their quality by removing some of the non-significant and entangled effects. The evaluation of statistical analysis tools like ANOVA for each response was used to determine the significance of each method parameter selected for the study using the p-value (significance level *p* < 0.05). For all models, good fits were obtained; coefficients of determination R^2^ were above 0.99, and lack of fits is not statistically significant (*p* > 0.05).

The results are evaluated based on the desirability factor used in the STATISTICA software [55,56,57]. The desirability factor approach is a very useful method for the optimization of multiple response processes. The relationship between the approximated (predicted) baseline responses and the utility of the response is called the desirability function. The idea behind this approach is the “quality” of a product or process that has multiple quality characteristics, with one of them outside of some “desirability” limits, is completely unacceptable. The method finds operating conditions that provide the “most desirability” response values. 0.0 represents a completely undesirable value and 1.0 represents a completely desirable or ideal response value. In this study, for resolutions between all pairs of peaks, desirability equals 0.0 when all resolution factors are less than 2.0, for symmetry factors desirability equals 1.0 when all symmetry factors are more than 0.8, and desirability equals 0.0 when all symmetry factors are more than 1.8.

Cumulative desirability plots of results and multiple response curves are used to identify the optimum pH of the aqueous mobile phase, range of start and end % of acetonitrile, and a suitable column for further optimization.

**Figure 4:**
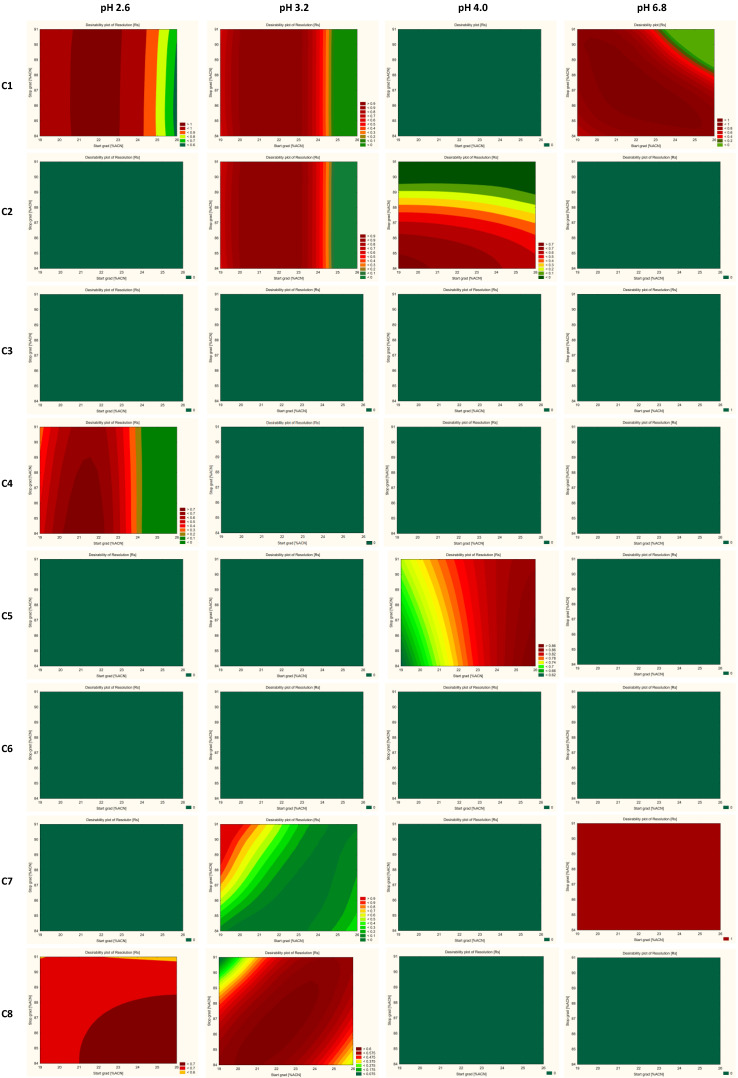
Screening experiment results showing the interaction effect of columns, % of organic solvent at the start and end gradient, and pH with CMAs as a desirability plot of resolutions (R_S_). C1—ZORBAX Eclipse Plus C18 (2.1 × 50 mm, 1.8 µm, Agilent Technologies), C2—Kinetex EVO C18 (2.1 × 50 mm, 1.7 µm, Phenomenex), C3—ACQUITY UPLC BEH C18 (2.1 × 50 mm, 1.7 µm Waters), C4—InfinityLab Poroshell 120 Phenyl Hexyl (2.1 × 50 mm, 1.9 µm, Agilent Technologies), C5—Kinetex Biphenyl (2.1 × 50 mm, 1.7 µm, Phenomenex), C6—Kinetex PFP (2.1 × 50 mm, 1.7 µm, Phenomenex), C7—ACQUITY UPLC CSH C18 (2.1 × 50 mm, 1.7 µm, Waters), and C8—ACQUITY UPLC CSH Fluoro-Phenyl (2.1 × 100 mm, 1.7 µm, Waters).

**Figure 5:**
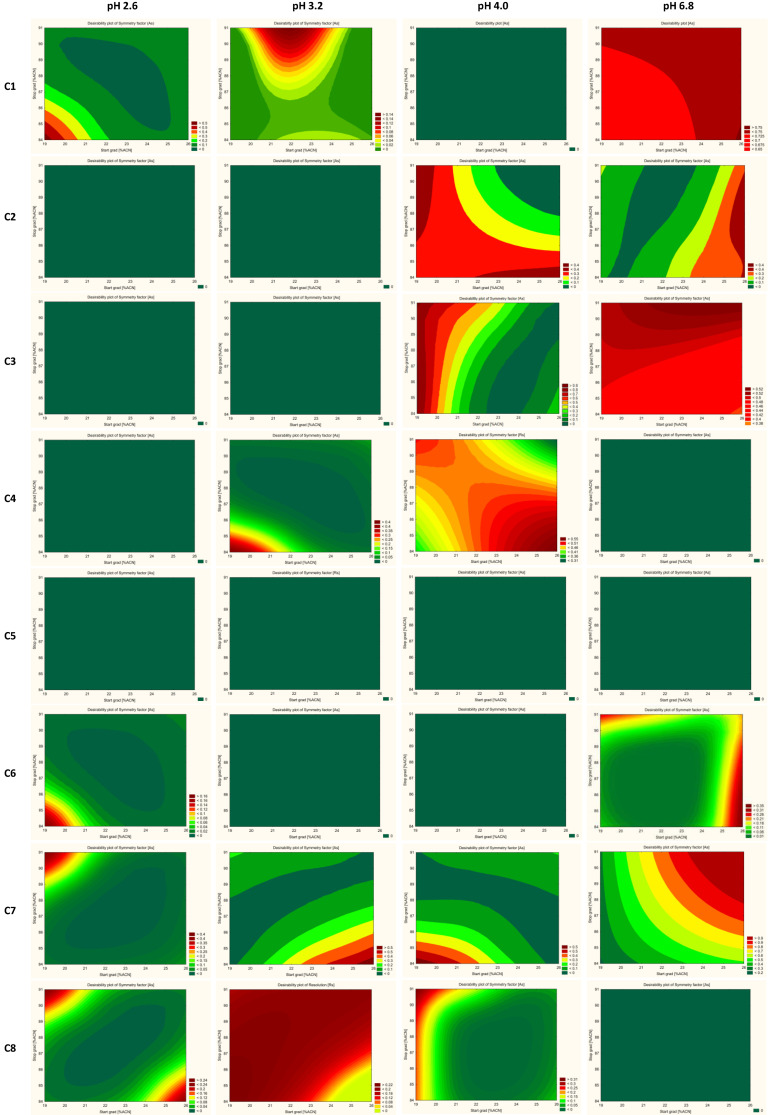
Screening experiment results showing the interaction effect of columns, % of organic solvent at the start and end gradient, and pH with CMAs as a desirability plot of symmetry factors (A_S_). C1—ZORBAX Eclipse Plus C18 (2.1 × 50 mm, 1.8 µm, Agilent Technologies), C2—Kinetex EVO C18 (2.1 × 50 mm, 1.7 µm, Phenomenex), C3—ACQUITY UPLC BEH C18 (2.1 × 50 mm, 1.7 µm Waters), C4—InfinityLab Poroshell 120 Phenyl Hexyl (2.1 × 50 mm, 1.9 µm, Agilent Technologies), C5—Kinetex Biphenyl (2.1 × 50 mm, 1.7 µm, Phenomenex), C6—Kinetex PFP (2.1 × 50 mm, 1.7 µm, Phenomenex), C7—ACQUITY UPLC CSH C18 (2.1 × 50 mm, 1.7 µm, Waters), and C8—ACQUITY UPLC CSH Fluoro-Phenyl (2.1 × 100 mm, 1.7 µm, Waters).

**Figure 6:**
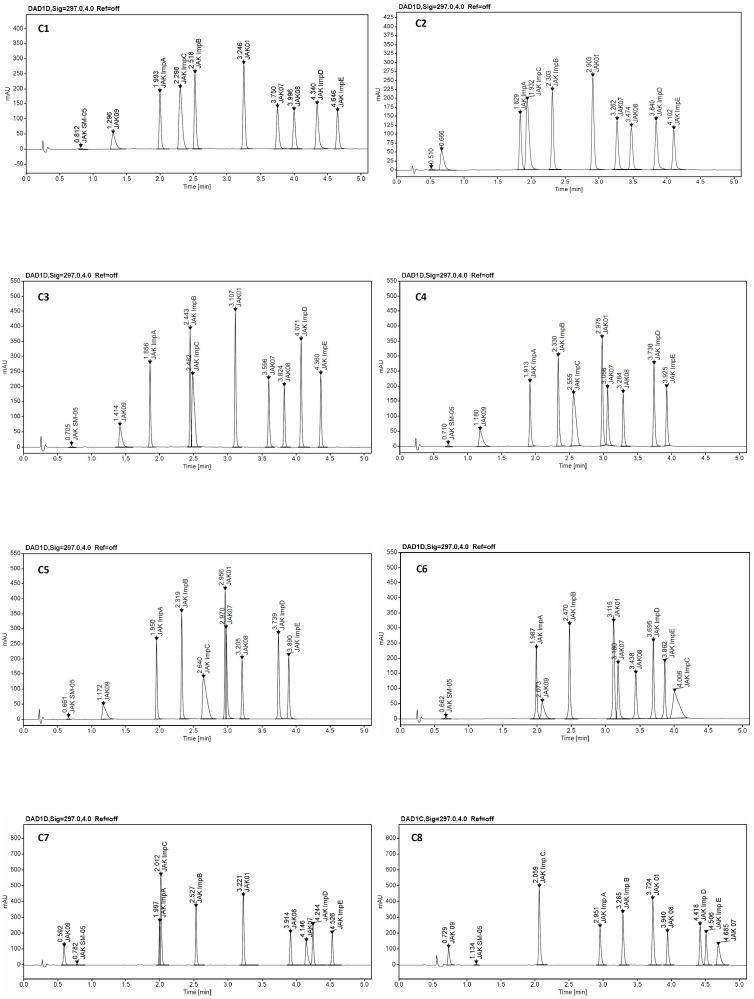
An example of chromatograms from screening experiments on the eight tested columns: C1—ZORBAX Eclipse Plus C18 (2.1 × 50 mm, 1.8 µm, Agilent Technologies), C2—Kinetex EVO C18 (2.1 × 50 mm, 1.7 µm, Phenomenex), C3—ACQUITY UPLC BEH C18 (2.1 × 50 mm, 1.7 µm Waters), C4—InfinityLab Poroshell 120 Phenyl Hexyl (2.1 × 50 mm, 1.9 µm, Agilent Technologies), C5—Kinetex Biphenyl (2.1 × 50 mm, 1.7 µm, Phenomenex), C6—Kinetex PFP (2.1 × 50 mm, 1.7 µm, Phenomenex), C7—ACQUITY UPLC CSH C18 (2.1 × 50 mm, 1.7 µm, Waters), and C8—ACQUITY UPLC CSH Fluoro-Phenyl (2.1 × 100 mm, 1.7 µm, Waters).

The trellis graph (Figure 4 and Figure 5) shows the results (desirability plot) of screening experiments using different columns, different pH, different start, and final % of acetonitrile, separately for the resolutions and symmetry factors. The red tint in the graphs shows the “knowledge space”, the area where all the critical method attributes (CMAs), i.e., the resolution between peaks, and symmetry factors are within the expected range (R_s_ ≥ 2.0, and 0.8 ≤ A_s_ ≤ 1.8), and green tint shows the area where no requirements of CMAs are not met. Figure 6 presented an example of chromatograms corresponding to the selected conditions of separation from screening tests.

Based on the evaluation of the screening experiments results calculated by the STATISTICA Software (version 13.3) TIBCO Inc. (Palo Alto, CA, USA), the values of the critical parameters of the methods (CMPs) allow for obtaining the optimal conditions, meeting the acceptance criteria (CMAs) for the separation of the test substance CPL409116, and its nine impurities are shown in Table 4.

For further study follow chromatographic condition was selected ZORBAX Eclipse Plus C18, pH 2.6, 20% of ACN at start gradient, 85% of ACN at end gradient; Kinetex EVO C18, pH 4.0, 21% of ACN at start gradient, 86% of ACN at end gradient; ACQUITY UPLC CSH Fluoro-Phenyl, pH 2.6, 20% of ACN at start gradient, 85% of ACN at end gradient.

### 2.5. Optimization and Robustness Testing

**Figure 7:**
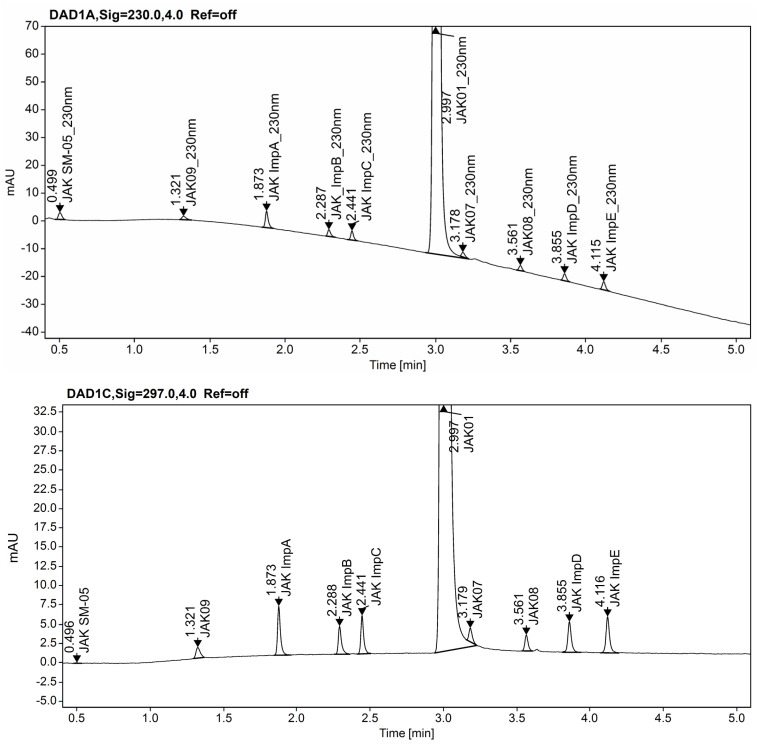
Chromatogram of JAK01 substance solution spiked with impurities at 0.15% level on the Kinetex EVO C18 (2.1 × 50 mm, 1.7 µm, Phenomenex) column.

In the following step of method development, the conditions chosen during the screening experiments were tested. The real sample of JAK01 substance and a sample of the test substance spiked with impurities at the 0.15% level, apart from the SST solution, were running, and robustness tests were performed. Although the acceptance criteria of CMAs were met for all three conditions selected for the screening study, a chromatogram of the real substance with overload concentrations of JAK01 with impurities spiked at 0.15% level is insufficient. On column Kinetex Evo C18, impurity JAK07 elutes on the slope of the main peak of JAK01 (Figure 7). On the ACQUITY UPLC CSH Fluoro-Phenyl column we observe a similar situation, impurity JAK08 elutes on the slope of the peak of JAK01. Because of this, there may be problems with coelution and peak integration when applying the method to routine testing.

Therefore, the best choice of chromatographic conditions for the determination of JAK01 impurities seems to be on the Zorbax Eclipse Plus C18 column, pH 2.6, with 20% of ACN at the start and 85% of ACN at the ending gradient. The higher resolution (R_s_ > 8.5) between the main peak of JAK01 and the nearest impurity is observed under this condition on the SST solution, compared to Kinetex Evo C18 and ACQUITY UPLC CSH Fluoro-Phenyl columns. On the Kinetex Evo C18 column, the resolution between JAK01 and the next peak JAK07 equals 2.2, and on the ACQUITY UPLC CSH Fluoro-Phenyl column resolution between JAK01 and JAK08 equals 5.1.

In the next step, separations conditions on a Zorbax Eclipse Plus C18 column with 10 mM formic acid pH 2.6 as an aqueous mobile phase were applied to the robustness test.

Typically, the robustness of a developed method is tested by changing one method parameter at a time, keeping the other variables constant. During our studies, many variables were changed simultaneously in various combinations using the DOE approach, based on the fractional factorial design with central point values, and with full repetition for statistical analysis of robustness.

Apart from variables tested at the screening step, the column temperature, and concentration of formic acid were additionally tested. The range of variable values was established from the knowledge space of the screening step, % of ACN at the start of the gradient (20% ± 1%), % of ACN at the end of the gradient (85% ± 1%). The concentration of formic acid was tested in the range of 10mM ± 1 mM, and the column temperature was 30 °C ± 2 °C. The other parameters of separation are kept at a constant value that was detailed described in Section 3.2.

The application of fractional factorial design (2^(4−1)^) with a center point and repetition instead of full factorial design with center points (3^4^) and repetition allows obtaining the same knowledge about the robustness of the method when performing 18 experiments instead of 81. The runs of the experimental plans were carried out in a randomized order with SST solution and a test solution containing 0.5 mg mL^−1^ of JAK01 and 0.75 µg mL^−1^ of JAK01 impurities corresponding to 0.15% level, to assure sufficient selectivity.

The design of the experiment plan and corresponding raw data were presented in Table 5.

**Figure 8:**
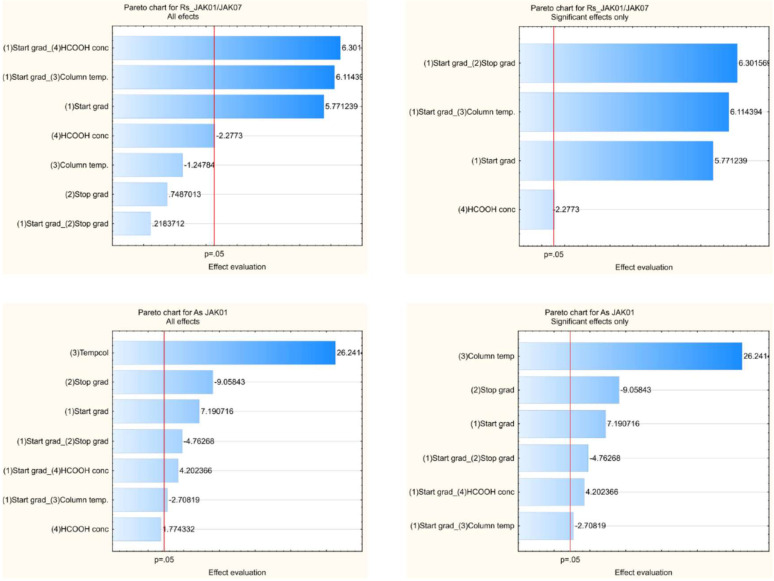
The Pareto chart for Rs between JAK01 and JAK07 and As for JAK01 for robustness tests.

Multiple linear regression was applied for the calculation of the coefficients of the nine models for resolution between all pairs of peaks and ten models of the symmetry factors for all peaks, then the models were refined to improve their quality by removing some of the non-significant and entangled effects. The evaluation of statistical analysis tools like ANOVA for each response was used to determine the significance of each method parameter selected for the study using the p-value (significance level *p* < 0.05). The graphical analysis of the effects presents in Figure 8 as an example of the Pareto chart (for R_s_ between JAK01 and JAK07 and A_s_ for JAK01), which allows the retained coefficients and the significant terms of the models to be identified the impact of the tested variables on the CMAs.

After verifying the reliability of the regression models, the results were converted to the desirability plots for a specified value of CMAs (R_s_ ≥ 2.0 for all pairs of peaks, and the symmetry factor of all peaks 0.8 ≤ A_s_ ≤ 1.8) for values of the CMPs from the robustness testing range. The analysis of these plots was used to estimate the final design space of the proposed method.

Based on the results of the statistical analysis, we can conclude that the assumed requirements for CQAs are met in the whole robustness tested range of the CMPs—desirability factors are well above 0.0, near the maximum value equals 1.0.

### 2.6. Method Operable Design Region (MODR) and Control Strategy

Based on the results of the design of the experiment from robustness tests, the STATISTICA software was also employed for verifying the MODR, i.e., it was checked whether the CMPs selected in the screening phase, in a small range centered on the optimized value, had a significant effect on CMAs.

For this purpose, probability maps were calculated by Monte-Carlo simulations, propagating the predictive error by using the model equation to the CMAs and computing the probability of reaching the desired objectives [11,12]. The threshold for the risk of failure was set to 10%. This means that in the calculated zone the values of the CMAs are satisfied with a probability of 90%. For all variables (CMAs) risk of failure was well below 10% at the robustness tested range of CMPs.

Based on the statistical result evaluations of the screening, optimization, robustness, and risk tests, the MODR corresponds to the following intervals, 20% ± 1% of ACN at the start of the gradient, 85% ± 1% of ACN at the end of the gradient, the concentration of formic acid 10 mM ± 1 mM, and the column temperature 30 °C ± 2 °C.

The control strategy of the method was designed by taking into account the results of robustness testing and identifying system suitability criteria [21]. The intervals for the accepted CMAs values were included between the lowest and the highest values for the CMAs measured when performing a robustness study. The obtained interval resolutions for the SST solution were shown in Table 5. Additionally, based on the screening study, we could define the requirements for the resolution between JAK01 and the nearest impurity peak for SST solution as more than 6.4. Less value of it causes that impurity peak may coelute with the main peak of JAK01 at an overloaded concentration in the test solution.

### 2.7. Validation

Validation of the method was carried out in compliance with ICH guideline Q2(R1) [25]. The validation data are reported in Table 6, showing adequate performances for the intended purpose. The determined content of all CPL409116 (JAK01) impurities showed evidence of good precision (RSDs were less than 10.0%). LOQ concentration value is on the reporting threshold level of 0.05% for all impurities.

**Figure 9:**
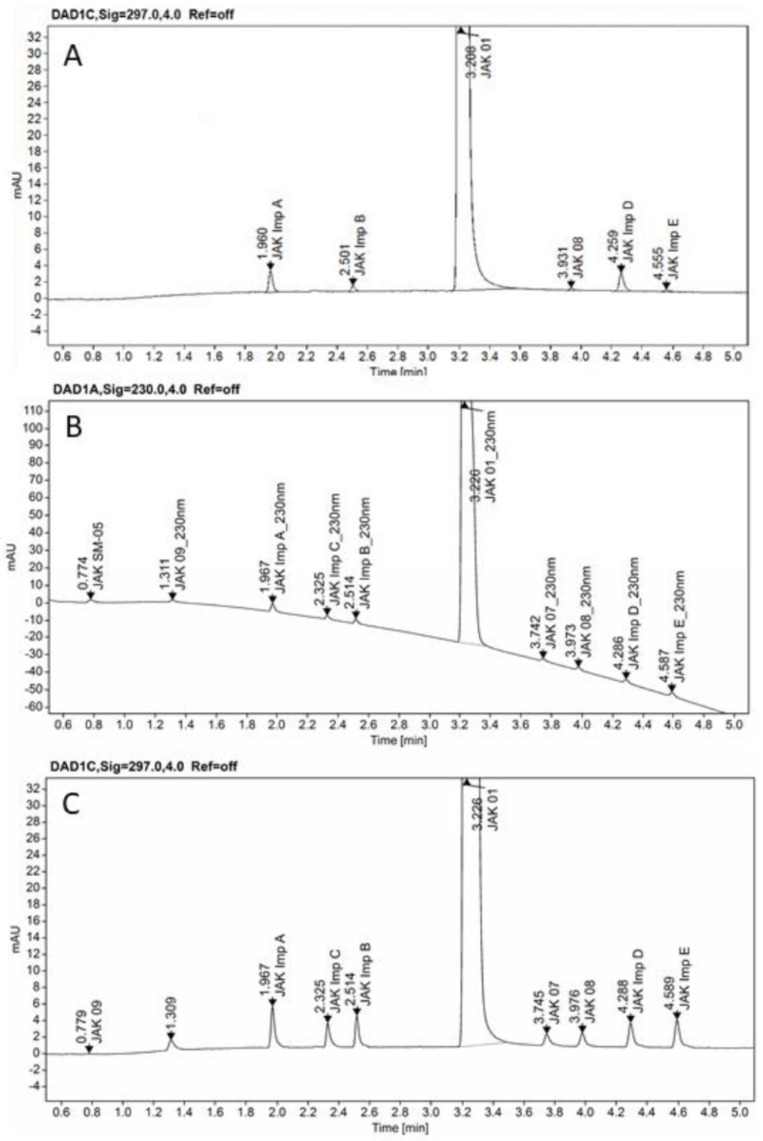
Overlay chromatograms JAK01 test solution (**A**), and JAK01 test solution spiked with impurities at 0.15% level at 230 nm (**B**), and at 297 nm (**C**), on the ZORBAX Eclipse Plus C18 (2.1 × 50 mm, 1.8 µm, Agilent Technologies, Waldbronn, Germany) column.

The developed method was finally applied for the analysis of a real sample of CPL409116 substance from a large-scale synthetic process. Overlay chromatograms JAK01 test solution and JAK01 test solution spiked with impurities at 0.15% level shown in Figure 9.

## 3. Material and Methods

### 3.1. Chemicals and Reagents

Reference standard JAK01 (CPL409116) and its impurities (JAK07, JAK08, JAK09, JAK ImpA, JAK ImpB, JAK ImpC, JAK ImpD, JAK ImpE, and JAK SM-05) were manufactured in-house by Celon Pharma S.A. (Lomianki, Poland).

Mix reference substances to the distribution coefficient chromatographically determinations (CHI logD) including paracetamol, acetanilide, acetophenone, propiophenone, butyrophenone, and valerophenone (purity >99.0%) were purchased from Sigma-Aldrich Chemie GmbH (Steinheim, Germany).

Acetonitrile (ACN) (hypergrade for LC-MS), and methanol (MeOH, hypergrade for LC-MS) were purchased from Merck KGaA (Darmstadt, Germany), and dimethyl sulfoxide (DMSO, for HPLC) from POCH (Gliwice, Poland). Formic acid (98–100%, eluent additive for LC-MS) and ammonia (25% solution, eluent additive for LC-MS) were obtained from CHEM-LAB NV (Zedelgem, Belgium). Ultra-pure water for HPLC was obtained from the water purification system Milli-Q IQ 7000 from Merck KGaA (Darmstadt, Germany).

### 3.2. Solutions and Sample Preparations

A number of buffers (10mM HCOONH_4_) with different pH levels were prepared for these experiments. Buffers (pH 3.2, 3.8, 4.0, 4.2, and 6.8) were prepared by mixing the formic acid solution with different levels of ammonia (25% solution) to adjust the pH. The individual phases of formic acid were prepared by adding a suitable amount of concentrated acid to water. Stock solutions were prepared for all compounds and stored at 4 °C. A standard stock solution of JAK01 (3 mg mL^−1^) was prepared using a mixture of DMSO:MeOH (20:80 *v*/*v*). Stock solutions (4 mg mL^−1^) for each impurity (JAK07, JAK08, JAK09, JAK ImpA, JAK ImpB, JAK ImpC, and JAK SM-05) were prepared in a mixture of DMSO: MeOH (20:80 *v*/*v*). Stock solutions (4 mg mL^−1^) for JAK ImpD and JAK ImpE were prepared in DMSO. Working standard solutions were daily prepared using MeOH. A mix of all compounds at the concentration level of 0.1–1 µM (as SST solution) was used to screen the chromatographic conditions. In the next stage of the research, a mixture of JAK01 (0.5 mg mL^−1^; 100%) with nine of its impurities (each at the level of 0.75 ug mL^−1^; 0.15%) was used. For the tested impurities, linearity in the range of 0.05% to 0.18% was also demonstrated by preparing mixtures of compounds at the appropriate concentration level and a solution of impurities at 0.02% was prepared for detection limit (LOD) determination.

For the CHI logD determination, separate solutions of all the tested sample were prepared at a concentration of 1 mM in DMSO. Reference mix solutions have a concentration of 1 mM in DMSO.

### 3.3. Instrumentations and Chromatographic Conditions

Eight different chromatographic columns were evaluated: ZORBAX Eclipse Plus C18 (2.1 × 50 mm, 1.8 µm, Agilent Technologies), Kinetex EVO C18 (2.1 × 50 mm, 1.7 µm, Phenomenex), ACQUITY UPLC BEH C18 (2.1 × 50 mm, 1.7 µm Waters), InfinityLab Poroshell 120 Phenyl Hexyl (2.1 × 50 mm, 1.9 µm, Agilent Technologies), Kinetex Biphenyl (2.1 × 50 mm, 1.7 µm, Phenomenex), Kinetex PFP (2.1 × 50 mm, 1.7 µm, Phenomenex), ACQUITY UPLC CSH C18 (2.1 × 50 mm, 1.7 µm, Waters), and ACQUITY UPLC CSH Fluoro-Phenyl (2.1 × 100 mm, 1.7 µm, Waters). When performing the chromatographic optimization, eluent A consisted of formic acid (10 mM, pH 2.6) and ammonium formate (10 mM) buffer with different concentrations of additives was used to prepare buffer solutions of 3.2, 4.0, and 6.8, and eluent B consisted of the organic solvent (ACN). The elution was performed with linear gradient mode. The starting and ending percentage of eluent B was optimized at a screening phase at a range from 20% to 25% and from 85% to 90%. The ending percentage of eluent B was isocratically maintained for 2 min. The whole gradient time was 7 min and included 4 min of elution step, 1 min of washing and 2 min of stabilization step. The other working conditions were as follows: sample injection volume, 1 µL; flow rate, 0.50 mL min^− 1^; oven temperature, 30 °C (28–32 °C); detection UV 297 nm (230 nm for JAK SM-05), and the autosampler temperature, 15 °C.

The chromatographic analyses were run by an Inifinity II 1290 UHPLC Method Development System (Agilent Technologies, Waldbronn, Germany) equipped with quaternary high-pressure pump, 12-position solvent selection valve, multisampler with thermostat, multicolumn thermostat including 8-position quick-change column selection valve and diode array detector. UHPLC system was controlled by OpenLab Client/Server System (version 2.5) (Agilent Technologies, Waldbronn, Germany).

The CHI logD for JAK01 and its impurities was determined on an UltraCore 2.5 SuperC18 (2.1 × 50 mm, 2.5 µm, ACE) column with eluent A consisting of formic acid (10 mM, pH 2.6), and ammonium acetate (50 mM) buffers with different concentrations of additives were used to prepare buffer solutions of 7.4, and 10.5, and eluent B consisted ACN [58].

### 3.4. Calculations and Software

Calculations of physico-chemical properties were made with the ACD Labs Percepta software (2020.1.1 release) (Advanced Chemistry Development, Inc., Toronto, ON, Canada) including PhysChem and Drug Profiler modules [40].

For evaluation of potential mutagenicity two complementary (Q)SAR prediction methodologies were used DEREK Nexus module (Lhasa Ltd., Leeds, UK) in the Star Drop software (version 6.6) (Optibrium Ltd., Cambridge, UK) [50,51,52] was used for rule-based calculations. For statistically-based (Q)SAR methodology the Toxicity Estimation Software Tool T.E.S.T (version 4.2.1) (US Environmental Protection Agency, Cincinnati, OH, USA) [53,54] software was used.

The STATISTICA (version 13.3) TIBCO Software Inc. (Palo Alto, CA, USA) was used for DOE and investigations—generate plans of the experiment, screening, optimization, robustness, and risk study of analytical method conditions, and statistical analysis of obtained results [55,56,57].

## 4. Conclusions

The present work has demonstrated the application of AQbD for the selection and development of a chromatographic analytical method for the determination of purity CPL409116 (JAK01) for controlling the large-scale synthetic process of the substance, performing stability tests, and manufacturing of the final drug form for clinical trials.

The ultimate goal of the chromatographic method development is to obtain an acceptable resolution of all components within a reasonable analysis time. To meet the quality requirements of the formal ICH regulatory guidelines [2,25], but bearing in mind the cost and time effectiveness of the preclinical stage of drug development, the main goal was to design a quick, simple, and robust analytical method.

At the beginning of the method development process, physico-chemical properties of CPL409116 and its related substances have been performed. In practice, not all values obtained from calculations give adequate information to use for chromatography method development. However, for this purpose, a very useful strategy is the determination of the hydrophobicity index CHI logD that gives also the possibility to narrow the scope of tested pH of the stationary phases at the screening stage.

In the present work, final method selection has been performed by screening analysis with eight chromatographic columns differing in chemical modifications of the stationary phase (C18, phenyl-hexyl, biphenyl), three linear gradients with various increasing of the organic phase, and aqueous phases differing in pH in acidic range (pH 2.6–6.8).

As a result, we selected three different conditions that meet acceptance criteria (ATP), but as a most universal condition, Agilent Zorbax Eclipse Plus C18 column with 10 mM formic acid pH 2.6 as an aqueous mobile phase, was applied for the robustness test.

Using a DOE approach and application of fractional factorial design with a center point and repetition, instead of full factorial design with center points and repetition, allows for obtaining the same knowledge about the robustness but is limited to 18 experiments based on simultaneous changes of many variables.

In this way, the need to perform a large number of analytical runs was effectively limited, confirming the scope of applicability of the selected analytical method with the AQbD method. Based on the results of the statistical analysis, we can conclude that the assumed requirements for CQAs are met in the whole robustness tested range of the CMPs—desirability factors are well above 0.0, near the maximum value equals 1.0.

## Figures and Tables

**Table 1:** Calculated physico-chemical properties and determined the chromatographic hydrophobicity index CHI logD of CPL409116 and its impurities.

Compound Name	logP	clogD 2.6	clogD 4.5	clogD 7.4	clogD 10.5	pKa (Acid)	pKa (Base)	CHILogD pH 2.6	CHILogD pH 7.4	CHILogD pH 10.5
**SM-05**	0.46	0.46	0.46	0.46	0.46	−	−2.50	−0.12	−0.03	0.99
**JAK-09**	1.27	−2.03	−1.83	−1.16	1.16	12.52	−1.12 2.68 9.92	0.71	1.22	2.38
**JAK ImpC**	−4.22	−4.29	−4.22	−4.22	−4.22	13.41	−0.95 2.00	1.25	3.41	3.23
**JAK ImpA**	0.54	0.1	0.53	0.43	−1.91	7.95 12.58	−0.53 2.00 2.68	1.49	1.49	1.99
**JAK ImpB**	1.07	−0.26	1.02	1.07	1.07	12.52 15.09	−1.11 2.70 3.56	2.40	2.21	2.45
**CPL409116** **(JAK01)**	1.44	0.98	1.43	1.44	1.44	12.52	−1.11 2.00 2.70	3.51	3.09	3.05
**JAK-07**	3.45	0.96	0.62	0.3	0.3	−	−0.78 0.90 3.98	3.80	2.55	2.57
**JAK-08**	2.55	2.3	2.55	2.55	2.54	12.52	−1.12 0.79 2.70	4.63	4.04	3.61
**JAK ImpE**	3	2.71	3	3	3	−	−1.20 1.98 2.40	4.86	4.82	4.07
**JAK ImpD**	2.65	2.36	2.65	2.65	2.65	−	−1.20 1.99 2.40	5.12	4.46	3.83

**Table 2:** List of the tested column and their properties: Fs—factor describes column similarity; H—parameter is a measure of the phase hydrophobicity; S—a measure of the resistance of the stationary phase to penetration by a solute molecule; A—measure of the hydrogen-bond acidity of the phase; B—a measure of the hydrogen-bond basicity of the phase; C—a measure of the interaction of the phase with ionized solute molecules.

L.p.	Column Name	Fs	H	S	A	B	C (pH 2.8)	C (pH 7.0)	EB	USP Type	Phase Chemistry
1.	ZORBAX Eclipse Plus C18	0	1.03	0	−0.0700	−0.0200	0	0.0200	7.8	L1	C18
2.	Kinetex EVO C18	9.65	1.01	−0.00600	−0.170	−0.0240	−0.110	−0.0100	4.38	L1	C18
3.	ACQUITY UPLC BEH C18	15.51	1.00	0.0280	−0.366	0.00700	0.142	0.0880	6.4	L1	C18
4.	InfinityLab Poroshell 120 Phenyl Hexyl	18.23	0.752	−0.0830	−0.394	0.0180	0.136	0.140	3.59	L11	Phenyl−Hexyl
5.	Kinetex Biphenyl	26.75	0.697	−0.173	−0.583	0.0340	0.122	0.817	2.63	L11	Biphenyl
6.	Kinetex PFP	78.79	0.680	0.0800	−0.270	−0.0300	0.940	1.53	2.4	L43	Pentafluorophenyl
7.	ACQUITY UPLC CSH C18	21.15	0.954	−0.00200	−0.179	0.118	0.0820	0.171	5.62	L1	C18
8.	ACQUITY UPLC CSH Fluoro−Phenyl	25.63	0.708	−0.0590	−0.435	0.129	−0.0680	0.223	2.865	L11	Fluoro−Phenyl

**Table 3:** Summary of evaluation of mutagenicity. (Q)SAR results for CPL409116 and related substances.

Compound Name	DEREK Nexus Results (Q)SAR Rule-Based	T.E.S.T Results (Q)SAR Statistic-Based	ICH M7 Class
**CPL409116** **(JAK-01)**	Mutagenicity is INACTIVE	Mutagenicity NEGATIVE Predicted value *p* = 0.25	Class 5
**JAK-07**	Mutagenicity is INACTIVE	Mutagenicity NEGATIVE Predicted value *p* = 0.26	Class 5
**JAK-08**	Mutagenicity is INACTIVE	Mutagenicity NEGATIVE Predicted value *p* = 0.23	Class 5
**JAK-09**	Mutagenicity is INACTIVE	Mutagenicity NEGATIVE Predicted value *p* = 0.39	Class 5
**JAK SM-5**	Mutagenicity is INACTIVE	Mutagenicity NEGATIVE Predicted value *p* = 0.22	Class 5
**JAK ImpA**	Mutagenicity is INACTIVE	Mutagenicity NEGATIVE Predicted value *p* = 0.16	Class 5
**JAK ImpB**	Mutagenicity is INACTIVE	Mutagenicity NEGATIVE Predicted value *p* = 0.28	Class 5
**JAK ImpC**	Mutagenicity is INACTIVE	Mutagenicity NEGATIVE Predicted value *p* = 0.29	Class 5
**JAK ImpD**	Mutagenicity is INACTIVE	Mutagenicity NEGATIVE Predicted value *p* = 0.15	Class 5
**JAK ImpE**	Mutagenicity is INACTIVE	Mutagenicity NEGATIVE Predicted value *p* = 0.16	Class 5

**Table 4:** Summary of the screening experiments results.

Column	pH of Buffer	Critical Method Parameters (CMAs)	Optimal Conditions of CMPs	
			Start Gradient [% ACN]	End Gradient [% ACN]
**ZORBAX Eclipse Plus C18** **(2.1 × 50 mm, 1.8 µm, Agilent Technologies)**	2.6	Symmetry factor of JAK09 and JAK ImpD	19–21	84–86
	3.2	Resolution of JAK SM-05/JAK09 Symmetry factor of JAK 09 Symmetry factor of JAK ImpC (>1.77) Symmetry factor of JAK ImpD	21–23	88–90
	4.0	Resolution of JAK ImpC/JAK ImpB (<1.5) Symmetry factor of JAK ImpD (>2.0) Symmetry factor of JAK ImpE (>1.9)	not found	not found
	6.8	Resolution of JAK ImpC—JAK Imp E	20–23	85–90
**Kinetex EVO C18** **(2.1 × 50 mm, 1.7 µm, Phenomenex)**	2.6	Resolution of JAK ImpA/JAK ImpB (<1.5) Symmetry factor of JAK09 (>1.8) Symmetry factor of JAK ImpC (>1.8)	not found	not found
	3.2	Symmetry factor of JAK09 (>1.8) Symmetry factor of JAK ImpB (>1.8) Symmetry factor of JAK ImpD (>1.8)	not found	not found
	4.0	Symmetry factor of JAK09 (<1.7) Symmetry factor of JAK ImpC (<1.7)	20–24	85–87
	6.8	Resolution of JAK ImpB/JAK07	not found	not found
**ACQUITY UPLC BEH C18** **(2.1 × 50 mm, 1.7 µm Waters)**	2.6	Resolution of JAK ImpB/JAK ImpC (<0.8)	not found	not found
	3.2	Resolution of JAK ImpC/JAK ImpB (<0.5) Symmetry factor of JAK09 (>2.1) Symmetry factor of JAK ImpC (>1.9)	not found	not found
	4.0	Resolution of JAK ImpB/JAK ImpC (<0.8)	not found	not found
	6.8	Symmetry factor of JAK09 (>1.6)	20–25	85–90
**InfinityLab Poroshell 120 Phenyl Hexyl** **(2.1 × 50 mm, 1.9 µm, Agilent Technologies)**	2.6	Symmetry factor of JAK ImpC (>2.0)	not found	not found
	3.2	Resolution of JAK07/JAK08 (<0.5) Symmetry factor JAK07 (>1.8)	not found	not found
	4.0	Resolution of JAK08/JAK01 (<0.5) Symmetry factor JAK07 (<0.6)	not found	not found
	6.8	Symmetry factor JAK09 (>2.0) Symmetry factor JAK07 (>1.8)	not found	not found
**Kinetex Biphenyl** **(2.1 × 50 mm, 1.7 µm, Phenomenex)**	2.6	Resolution of JAK07/JAK01 (<0.6) Symmetry factor of JAK09 (>2.3) Symmetry factor of JAK ImpB (>2.4) Symmetry factor of JAK07 (>2.3)	not found	not found
	3.2	Resolution of JAK07/JAK01 (<1.1) Symmetry factor of JAK09 (>1.9) Symmetry factor of JAK ImpB (>1.9)	not found	not found
	4.0	Symmetry factor of JAK01 (>2.5)	not found	not found
	6.8	Resolution of JAK07/JAK ImpB (<0.8) Symmetry factor of JAK07 (>2.4)	not found	not found
**Kinetex PFP** **(2.1 × 50 mm, 1.7 µm, Phenomenex)**	2.6	Resolution of JAK09/JAK ImpA (<1.6) Symmetry factor of JAK09 (>1.8) Symmetry factor of JAK ImpC (>1.8)	not found	not found
	3.2	Resolution of JAK08/JAK07 (<0.5) Symmetry factor of JAK09 (>1.8) Symmetry factor of JAK ImpC (>2.1)	not found	not found
	4.0	Resolution of JAK ImpE/JAK ImpD (<0.8) Symmetry factor of JAK ImpC (>1.9)	not found	not found
	6.8	Resolution of JAK08/JAK07 (<0.85)	not found	not found
**ACQUITY UPLC CSH C18** **(2.1 × 50 mm, 1.7 µm, Waters)**	2.6	Resolution of JAK ImpC/JAK ImpA (<1.8) Symmetry factor of JAK07 (>1.8)	not found	not found
	3.2	Resolution of JAK07/JAK08 (<2.0) Symmetry factor of JAK07 (>1.8)	not found	not found
	4.0	Resolution of JAK ImpB/JAK ImpC (<0.6)	not found	not found
	6.8	Symmetry factor of JAK09 Symmetry factor of JAK ImpB Symmetry factor of JAK07	20–23	85–87
**ACQUITY UPLC CSH** **Fluoro-Phenyl** **(2.1 × 100 mm, 1.7 µm, Waters)**	2.6	Resolution JAK ImpE/JAK ImpD (<2.28, >2.00) Symmetry factor of JAK07 (<1.8)	20–21	89–90
	3.2	Resolution JAK ImpE/JAK ImpD (<2.3, >2.00) Symmetry factor of JAK07 (<1.8)	20–23	85–90
	4.0	Resolution JAK07/JAK ImpE (<1.3)	not found	not found
	6.8	Resolution JAK08/JAK01 Symmetry factor of JAK01 (<0.8)	not found	not found

**Table 5:** Plan of the design and raw data of robustness experiments.

Start Gradient [%ACN]	End Gradient [%ACN]	Column Temperature [°C]	Concentration of HCOOH Solution [mM]	Results of R_s_										Results of A_s_								
				JAK 09	JAK Imp A	JAK Imp C	JAK Imp B	JAK 01	JAK 07	JAK 08	JAK Imp D	JAK Imp E	JAK SM-05	JAK 09	JAK Imp A	JAK Imp C	JAK Imp B	JAK 01	JAK 07	JAK 08	JAK Imp D	JAK Imp E
19.0	84.0	28.0	9.0	11.20	7.83	5.01	2.79	11.70	7.42	4.20	4.2	4.36	1.57	1.35	1.28	1.40	1.25	1.20	1.26	1.17	1.22	1.23
21.0	86.0	28.0	9.0	3.02	8.27	3.95	3.14	12.15	7.61	3.60	4.57	4.19	1.56	1.79	1.31	1.46	1.26	1.18	1.22	1.18	1.16	1.20
19.0	86.0	28.0	11.0	6.88	7.04	3.63	2.87	10.20	6.75	2.83	3.74	3.54	1.56	1.43	1.15	1.44	1.14	1.16	1.21	1.20	1.20	1.20
21.0	84.0	28.0	11.0	2.70	7.00	4.23	2.96	10.75	6.96	2.96	3.80	3.75	1.60	1.65	1.37	1.45	1.49	1.43	1.39	1.39	1.50	1.51
19.0	86.0	32.0	9.0	6.43	6.71	3.28	3.08	9.50	6.44	2.62	3.40	3.51	1.54	1.47	1.55	1.38	1.55	1.59	1.44	1.48	1.51	1.52
21.0	84.0	32.0	9.0	2.57	8.32	3.77	3.37	11.92	7.52	3.11	4.16	4.31	1.46	1.65	1.71	1.57	1.73	1.69	1.60	1.59	1.60	1.65
19.0	84.0	32.0	11.0	6.09	7.22	3.38	3.02	12.00	6.90	3.18	4.25	4.28	1.42	1.47	1.78	1.49	1.68	1.59	1.51	1.46	1.52	1.57
21.0	86.0	32.0	11.0	2.35	8.28	3.75	3.32	11.69	7.78	3.06	4.14	4.18	1.60	1.64	1.77	1.53	1.63	1.56	1.49	1.45	1.54	1.54
20.0	85.0	30.0	10.0	5.73	9.32	4.54	3.24	14.04	8.53	3.98	5.43	4.76	1.39	1.72	1.31	1.38	1.41	1.32	1.28	1.23	1.40	1.24
19.0	84.0	28.0	9.0	11.6	7.07	5.38	2.84	11.51	7.61	4.28	4.09	4.30	1.55	1.34	1.33	1.38	1.27	1.25	1.25	1.22	1.26	1.29
21.0	86.0	28.0	9.0	3.07	8.15	4.01	3.18	12.05	7.45	3.45	4.85	4.49	1.56	1.79	1.27	1.46	1.23	1.18	1.18	1.15	1.18	1.23
19.0	86.0	28.0	11.0	7.34	7.28	3.81	2.91	10.91	6.99	3.02	3.88	3.61	1.53	1.43	1.12	1.48	1.10	1.11	1.15	1.12	1.18	1.22
21.0	84.0	28.0	11.0	2.65	7.73	4.02	2.88	10.32	6.64	2.78	4.06	3.93	1.63	1.62	1.34	1.42	1.41	1.46	1.40	1.41	1.46	1.50
19.0	86.0	32.0	9.0	6.36	6.79	3.29	3.17	9.51	6.52	2.66	3.39	3.50	1.54	1.44	1.52	1.36	1.51	1.52	1.41	1.42	1.51	1.51
21.0	84.0	32.0	9.0	2.54	8.37	3.75	3.36	11.27	7.19	2.98	4.02	4.20	1.45	1.66	1.73	1.61	1.77	1.67	1.54	1.54	1.58	1.61
19.0	84.0	32.0	11.0	6.37	7.38	3.70	3.18	12.33	6.55	3.20	4.00	4.32	1.40	1.50	1.78	1.52	1.69	1.57	1.48	1.45	1.53	1.55
21.0	86.0	32.0	11.0	2.34	8.20	3.77	3.34	11.77	7.73	3.02	4.10	4.14	1.62	1.66	1.72	1.53	1.65	1.59	1.49	1.46	1.59	1.56
20.0	85.0	30.0	10.0	5.75	9.26	4.53	3.27	14.10	8.51	3.99	5.41	4.70	1.39	1.72	1.35	1.41	1.38	1.30	1.32	1.23	1.55	1.28
min				2.34	6.71	3.28	2.79	9.50	6.44	2.62	3.39	3.50	1.39	1.34	1.12	1.36	1.10	1.11	1.15	1.12	1.16	1.20
max				11.60	9.32	5.38	3.37	14.10	8.53	4.28	5.43	4.76	1.63	1.79	1.78	1.61	1.77	1.69	1.60	1.59	1.60	1.65

**Table 6:** Summary of the validation results.

Parameter	Acceptance Criteria	JAK SM-05	JAK 09	JAK Imp A	JAK Imp C	JAK Imp B	JAK 07	JAK 08	JAK Imp D	JAK Imp E	Compliance with Acceptance Criteria (Yes/No)
**Linearity, R**	LOQ—120% of the specification limit (for each impurity) Concentration levels *n* = 5, R ≥ 0.99	0.99	0.99	0.99	0.99	0.99	0.99	0.99	0.99	0.99	Yes
**Repeatability, RSD (%)**	LOQ—%RSD (area) ≤ 15 % 100% of the specification limit—%RSD (area) ≤ 15 %	6.8	8.5	2.7	4.7	4.2	9.1	3.4	2.5	2.0	Yes
		4.5	5.2	0.8	1.8	2.0	5.3	1.8	3.3	1.5	Yes
**Limit of Detection (LOD),**	S/N ≥ 3 (*n* = 3)	6	3	8	9	11	5	5	4	11	Yes
**Limit of Quantitation (LOQ)**	S/N ≥ 10 (*n* = 6)	21	12	22	29	31	14	16	8	32	Yes
**Specificity**	Resolution between two neighboring peaks, Rs ≥ 2.0	-	9.6	12.0	8.4	4.6	9.9	4.1	5.8	5.6	Yes

## Data Availability

Not applicable.

## References

[1] Elder D., Teasdale A. (2017). ICH, Q9, Quality Risk Management. ICH Quality Guidelines: An Implementation Guide.

[2] Holm P., Allesø M., Bryder M.C., Holm R. (2017). ICH, Q8(R2), Pharmaceutical Development. ICH Quality Guidelines: An Implementation Guide.

[3] Borman P., Chatfield M., Nethercote P., Thompson D., Truman K. (2007). The application of quality by design to analytical methods. Pharm. Technol..

[4] Schweitzer M., Pohl M., Brown M.H., Nethercote P., Borman P., Smith K., Larew J. (2010). Implications and Opportunities of Applying QbD Principles to Analytical Measurements. Pharm. Technol..

[5] Vogt F.G., Kord A.S. (2011). Development of quality-by-design analytical methods. J. Pharm. Sci..

[6] Castle B.C., Forbes R.A. (2013). Impact of Quality by Design in Process Development on the Analytical Control Strategy for a Small-Molecule Drug Substance. J. Pharm. Innov..

[7] Reid G., Morgado J., Barnett K., Harrington B., Harwood J., Fortin D. (2013). Analytical quality by design (AQbD) in pharmaceutical development. Am. Pharm. Rev..

[8] Parra M.K., Schmidt A.H. (2018). Life Cycle Management of Analytical Methods. J. Pharm. Biomed..

[9] Dispas A., Avohou H.T., Lebrun P., Hubert P., Hubert C. (2018). Quality by design approach for the analysis of impurities in pharmaceutical drug products and drug substances. TrAC Trends Anal. Chem..

[10] Basso J., Mendes M., Cova T.F., Sousa J.J., Pais A.A., Vitorino C. (2018). Analytical Quality by Design (AQbD) as a multiaddressable platform for co-encapsulating drug assays. The Royal Society of Chemistry 2018. Anal. Methods.

[11] Pasquini B., Orlandini S., Furlanetto S., Gotti R., Del Bubba M., Boscaro F., Bertaccini B., Douša M., Pieraccini G. (2020). Qualit by Design as risk-based strategy in pharmaceutical analysis: Development of a liquid chromatography-tandem mass spectrometry method for determination of nintedanib and its impurities. J. Chromatogr. A.

[12] Pawar A., Pandita N. (2020). Statistically Designed, Targeted Profile UPLC Method Development for Assay and Purity of Haloperidol in Haloperidol Drug Substance and Haloperidol 1 mg Tablets. Chromatographia.

[13] Hibbert D.B. (2012). Experimental design in chromatography: A tutorial review. J. Chromatogr. B.

[14] Kochling J., Wu W., Hua Y., Guan Q., Castaneda-Merced J. (2016). A platform analytical quality by design (AQbD) approach for multiple UHPLC-UV and UHPLC–MS methods development for protein analysis. J. Pharm. Biomed. Anal..

[15] Gaudin K., Ferey L. (2016). Quality by Design: A Tool for Separation Method Development in Pharmaceutical Laboratories. LCGC.

[16] Rácz N., Molnár I., Zöldhegyi A., Rieger H.-J., Kormány R. (2018). Simultaneous optimization of mobile phase composition and pH using retention modeling and experimental design. J. Pharm. Biomed. Anal..

[17] Orlandini S., Pinzauti S., Furlanetto S. (2013). Application of quality by design to the development of analytical separation methods. Anal. Bioanal. Chem..

[18] Li Y., Terfloth G.J., Kord A.S. (2009). A systematic approach to RP-HPLC method development in a pharmaceutical QbD environment. Am. Pharm. Rev..

[19] Bhaskaran N.A., Kumar L., Reddy M.S., Pai G.K. (2021). An analytical “quality by design” approach in RP-HPLC method development and validation for reliable and rapid estimation of irinotecan in an injectable formulation. Acta Pharm..

[20] Jackson P., Borman P.J., Campa C., Chatfield M.J., Godfrey M., Hamilton P.R., Hoyer W., Norelli F., Orr R., Schofield T. (2019). Using the analytical target profile to drive the analytical method lifecycle. Anal. Chem..

[21] Rathore A.S., Winkle H. (2009). Quality by design for biopharmaceuticals. Nat. Biotechnol..

[22] Yu L.X. (2007). Pharmaceutical quality by design: Product and process development, understanding, and control. Pharm. Res..

[23] Csoka I., Pallagi E., Paal T.L. (2018). Extension of quality-by-design concept to the early development phase of pharmaceutical R&D processes. Drug Discov. Today.

[24] Deidda R., Orlandini S., Hubert P., Hubert C. (2018). Risk-based approach for method development in pharmaceutical quality control context: A critical review. J. Pharm. Biomed. Anal..

[25] ICH Harmonised Tripartite Guideline. Validation of Analytical Procedures: Text and Methodology Q2(R1). Proceedings of the International Conference on Harmonisation of Technical Requirements for Registration of Pharmaceuticals for Human Use.

[26] Final Concept Paper: ICH Q14: Analytical Procedure Development and Revision of Q2(R1) Analytical Validation. https://www.ich.org/fileadmin/Public_Web_Site/ICH_Products/Guidelines/Quality/Q2_Q14/Q2R2Q14EWG_ConceptPaper_2018_1115.pdf.

[27] Tome T., Žigart N., Časar Z., Obreza A. (2019). Development and Optimization of Liquid Chromatography Analytical Methods by Using AQbD Principles: Overview and Recent Advances. Org. Process Res. Dev..

[28] Mroczkiewicz M., Stypik B., Bujak A., Szymczak K., Gunerka P., Dubiel K., Wieczorek M., Pieczykolan J. (2018). Pyrazole[1,5-a]Pyrimidine Derivatives as Kinase Jak Inhibitors. W.O. Patent.

[29] Mroczkiewicz M., Stypik B., Bujak A., Szymczak K., Gunerka P., Dubiel K., Wieczorek M., Pieczykolan J. (2020). Pyrazole[1,5-a]Pyrimidine Derivatives as Kinase Jak Inhibitors. E.P. Patent.

[30] Dulak-Lis M., Bujak A., Gala K., Banach M., Kędzierska U., Miszkiel J., Hucz-Kalitowska J., Mroczkiewicz M., Stypik B., Szymczak K. (2021). A novel JAK/ROCK inhibitor, CPL409116, demonstrates potent efficacy in the mouse model of systemic lupus erythematosus. J. Pharmacol. Sci..

[31] Iuliani P., Carlucci G., Marrone A. (2010). Investigation of the HPLC response of NSAIDs by fractional experimental design and multivariate regression analysis. Response optimization and new retention parameters. J. Pharm. Biomed. Anal..

[32] Kishore C.R.P., Mohan G.V.K. (2016). Development and Validation of Amlodipine Impurities in Amlodipine Tablets Using Design Space Computer Modeling. Am. J. Anal. Chem..

[33] Prasad S.S., Mohan G.V.K., Babu A.N. (2019). Orient. J. Chem..

[34] Sahu P.K., Ramisetti N.R., Cecchi T., Swain S., Patro C.S., Panda J. (2018). An overview of experimental designs in HPLC method development and validation. J. Pharm. Biomed. Anal..

[35] OECD (1995). Test No. 107: Partition Coefficient (n-Octanol/Water): Shake Flask Method.

[36] Albert A. (1979). Selective Toxicity: The Physicochemical Basis of Therapy.

[37] Klose M.H.M., Theiner S., Varbanov H.P., Hoefer D., Pichler V., Galanski M., Meier-Menches S.M., Keppler B.K. (2018). Development and Validation of Liquid Chromatography-Based Methods to Assess the Lipophilicity of Cytotoxic Platinum(IV) Complexes. Inorganics.

[38] Dolan J.W. (2017). Back to Basics: The Role of pH in Retention and Selectivity. LCGC N. Am..

[39] Lewis J.A., Lommen D.C., Raddatz W.D., Dolan J.W., Snyder L.R., Molnar I. (1992). Computer simulation for the prediction of separation as a function of pH for reversed-phase high-performance liquid chromatography: I. Accuracy of a theory-based model. J. Chromatogr. A.

[40] (2020). ACD/Percepta.

[41] Valkó K., Bevan C., Reynolds D. (1997). Chromatographic Hydrophobicity Index by Fast-Gradient RP-HPLC: A High-Throughput Alternative to log P/log D. Anal. Chem..

[42] Valko K. (2014). Physicochemical and Biomimetic Properties in Drug Discovery—Chromatographic Techniques for Lead Optimization.

[43] Snyder L.R., Dolan J.W., Carr P.W. (2004). The hydrophobic-subtraction model of reversed-phase column selectivity. J. Chromatogr. A.

[44] Snyder L.R., Dolan J.W., Marchand D.H., Carr P.W. (2012). The hydrophobic-subtraction model of reversed-phase column selectivity. Adv. Chromatogr..

[45] http://www.hplccolumns.org.

[46] https://apps.usp.org/app/USPNF/columnsDB.html.

[47] Committee for Human Medicinal Products (2015). ICH guideline M7(R1) on assessment and control of DNA reactive (mutagenic) impurities in pharmaceuticals to limit potential carcinogenic risk. Int. Conf. Harmon..

[48] Baertschi S., Olsen B., Riley C.M., Rosanske T.W., Reid G. (2020). Chapter 12—Mutagenic impurities. Specification of Drug Substances and Products.

[49] Shaikh T., Gosar A., Sayyed H. (2020). Nitrosamine Impurities in Drug Substances and Drug Products. J. Adv. Pharm. Pract..

[50] Orbitrium DEREK Nexus—Trial licence. https://www.optibrium.com.

[51] Lhasa Limited DEREK Nexus. https://www.lhasalimited.org.

[52] Marchant C.A., Briggs K.A., Long A. (2008). In Silico Tools for Sharing Data and Knowledge on Toxicity and Metabolism: Derek for Windows, Meteor, and Vitic. Toxicol. Mech. Methods.

[53] US EPA Research Toxicity Estimation Software Tool (TEST). https:/www.epa.gov/chemical-research/toxicity-estimation-software-tool-test.

[54] Martin T. (2016). User’s Guide for T.E.S.T. (Version 4.2) (Toxicity Estimation Software Tool) A Program to Estimate Toxicity from Molecular Structure.

[55] (2006). StatSoft’s Electronic Statistics Textbook. http://www.statsoft.pl/textbook/stathome.html.

[56] TIBCO Software Inc (2020). Data Science Textbook. https://docs.tibco.com/data-science/textbook.

[57] Stanisz A. (2006). Przystępny kurs Statystyki z Zastosowaniem STATISTICA PL na Przykładach z Medycyny—Tom I–III.

[58] Valko K.L. (2019). Application of biomimetic HPLC to estimate in vivo behavior of early drug discovery compounds. Future Drug Discov..

